# Copy number variations of genes involved in stress responses reflect the redox state and DNA damage in brewing yeasts

**DOI:** 10.1007/s12192-016-0710-8

**Published:** 2016-06-14

**Authors:** Jagoda Adamczyk, Anna Deregowska, Marek Skoneczny, Adrianna Skoneczna, Urszula Natkanska, Aleksandra Kwiatkowska, Ewa Rawska, Leszek Potocki, Ewelina Kuna, Anita Panek, Anna Lewinska, Maciej Wnuk

**Affiliations:** 1 Department of Genetics, University of Rzeszow, Rejtana 16C, 35-959 Rzeszow, Poland; 2 Postgraduate School of Molecular Medicine, Medical University of Warsaw, Warsaw, Poland; 3 Department of Genetics, Institute of Biochemistry and Biophysics, Polish Academy of Sciences, Warsaw, Poland; 4 Laboratory of Mutagenesis and DNA Repair, Institute of Biochemistry and Biophysics, Polish Academy of Sciences, Warsaw, Poland; 5 Department of Biochemistry and Cell Biology, University of Rzeszow, Zelwerowicza 4, 35-601 Rzeszow, Poland

**Keywords:** Brewing yeasts, Genome, Array-CGH, DNA damage, Redox equilibrium

## Abstract

The yeast strains of the *Saccharomyces* sensu stricto complex involved in beer production are a heterogeneous group whose genetic and genomic features are not adequately determined. Thus, the aim of the present study was to provide a genetic characterization of selected group of commercially available brewing yeasts both ale top-fermenting and lager bottom-fermenting strains. Molecular karyotyping revealed that the diversity of chromosome patterns and four strains with the most accented genetic variabilities were selected and subjected to genome-wide array-based comparative genomic hybridization (array-CGH) analysis. The differences in the gene copy number were found in five functional gene categories: (1) maltose metabolism and transport, (2) response to toxin, (3) siderophore transport, (4) cellular aldehyde metabolic process, and (5) L-iditol 2-dehydrogenase activity (*p* < 0.05). In the Saflager W-34/70 strain (Fermentis) with the most affected array-CGH profile, loss of aryl-alcohol dehydrogenase (*AAD*) gene dosage correlated with an imbalanced redox state, oxidative DNA damage and breaks, lower levels of nucleolar proteins Nop1 and Fob1, and diminished tolerance to fermentation-associated stress stimuli compared to other strains. We suggest that compromised stress response may not only promote oxidant-based changes in the nucleolus state that may affect fermentation performance but also provide novel directions for future strain improvement.

**Electronic supplementary material:**

The online version of this article (doi:10.1007/s12192-016-0710-8) contains supplementary material, which is available to authorized users.

## Introduction

Beer, one of the most common alcoholic beverages consumed worldwide, is produced by the fermentation of sugars into alcohol by the action of yeasts. Brewing yeasts are typically divided into two groups, ale brewing yeasts and lager brewing yeasts, according to their use for the production of ale and lager beers, respectively (Gibson and Liti 2015; Kodama et al. 2006; Wendland 2014). They differ in sugar utilization, flocculation, off-flavor production, and overall fermentation performance (Gibson and Liti 2015; Kodama et al. 2006; Wendland 2014). Ale yeasts are called top-fermenting yeasts because of their tendency to become buoyant after flocculation and rise to the surface of fermenting wort, whereas lager yeasts are cryotolerant and have a tendency to sediment after flocculation and sink to the bottom of the fermentation vessel and are named bottom-fermenting yeasts (Gibson and Liti 2015; Kodama et al. 2006; Wendland 2014). Ale beer has been produced for thousands of years and is usually brewed at 20–30 °C (Legras et al. 2007). However, nowadays, the more popular is lager beer that has been originated in the fifteenth century in Bavaria (Germany) and is usually brewed at low temperatures (5–15 °C) (Gibson and Liti 2015). The nomenclature of brewing yeasts is not consistent that also reflects their genomic diversity (Querol and Bond 2009). Ale strains of diploid nature are thought to be varieties of *Saccharomyces cerevisiae*, but also there are evidences that they may be hybrids, e.g., hybrids between *S. cerevisiae* and *Saccharomyces kudriavzevii* (Gonzalez et al. 2008; Querol and Bond 2009). Lager yeasts have been already classified as *Saccharomyces carlsbergensis*, *Saccharomyces pastorianus*, or *Saccharomyces uvarum*, but actually, they are not species of their own (Wendland 2014). They are interspecific hybrids between *S. cerevisiae* and the newly discovered and cold-tolerant *Saccharomyces eubayanus* (Caesar et al. 2007; Dunn and Sherlock 2008; Naumova et al. 2005). The *cerevisiae* part of lager yeast genome has been postulated to be an ale yeast, e.g., Fosters O-like ale yeast and/or stouts yeast (Dunn and Sherlock 2008; Monerawela et al. 2015). Lager yeasts can be further divided into two genetic groups (group I, Saaz/Carlsberg group and group II, Frohberg group) that differ in fermentation performance, sugar utilization and adaptation to growth at low temperature (Dunn and Sherlock 2008; Gibson et al. 2013). There are conflicting results on the ploidy state of these two groups of lager yeasts (Dunn and Sherlock 2008; Walther et al. 2014). Group I has been reported to be 2n or 3n and group II to be 3n or 4n (Dunn and Sherlock 2008; Querol and Bond 2009; Walther et al. 2014). However, more recently, it has been shown that *S. carlsbergensis* (group I) is essentially triploid, whereas Weihenstephan 34/70 strain (group II) is (allo)tetraploid (Walther et al. 2014). Aneuploidy and regions with copy number variations have been also revealed in lager yeasts (Bond et al. 2004). The hybrid and dynamic nature of lager yeast genome may provide adaptive potential but could also result in genomic instability adversely modulating fermentation performance and finally affecting the quality of beer. Thus, it seems worthwhile to monitor genetic and genomic features of brewing yeast strains, especially that their genomes are dynamic and may undergo rearrangements and gene amplification in response to stresses experienced during the brewing process (James et al. 2008).

In the present study, we have investigated the chromosome profiles of 30 industrial yeast strains used in brewing (*n* = 29) and cider production (*n* = 1) and subjected four strains with distinct chromosome patterns to array-based comparative genomic hybridization (array-CGH)-based genome-wide analysis. Regions with variations in gene copy number were revealed, and we found that aryl-alcohol dehydrogenase gene dosage correlated with intracellular redox equilibrium, genetic stability, and the nucleolar state, which may modulate tolerance to stress stimuli during fermentation conditions in brewing yeasts.

## Materials and methods

### Chemicals

All reagents were obtained from Sigma (Poznan, Poland) unless otherwise specified.

### Yeast strains and growth conditions

All brewing yeast strains used in this work are listed in Table 1.

**Table 1:** Brewing yeast strains used in this study

No.	Trade name	Company
1	Safale S-04	Fermentis
2	Safale US-05	Fermentis
3	Safbrew S-33	Fermentis
4	Safbrew T-58	Fermentis
5	Safbrew WB-06	Fermentis
6	Saflager W-34/70	Fermentis
7	Saflager S-23	Fermentis
8	Belle Saison Belgian Ale Yeast	Lallemand
9	Windsor British Ale Yeast	Lallemand
10	Munich German Wheat Beer Yeast	Lallemand
11	BRY-97 American West Coast Yeast	Lallemand
12	Gozdawa American West Coast Yeast	Gozdawa
13	Gozdawa Pure Ale Yeast 7 (PAY7)	Gozdawa
14	Gozdawa Porter & Kvass (POK V)	Gozdawa
15	Gozdawa Bavarian Wheat 11 (BW11)	Gozdawa
16	Gozdawa Old German Altbier 9 (OGA9)	Gozdawa
17	Gozdawa Classic Belgian Witbier (CBW)	Gozdawa
18	Gozdawa Czech Pilsner 18 (CP18)	Gozdawa
19	Mauribrew Ale Y514	Mauribrew
20	Mauribrew Weiss Y1433	Mauribrew
21	Coobra Allround Yeast	CBF Drinkit
22	Muntons Premium Gold Yeast	Muntons
23	Brewferm Top	Brewferm
24	Gozdawa Belgian Fruit and Spicy Ale Yeast (BFSAY)	Gozdawa
25	Gozdawa Fruit Blanche B1 (FBG1)	Gozdawa
26	Gozdawa French Cider G1 (FCG1)	Gozdawa
27	Brewferm Blanche	Brewferm
28	Wyeast 1056 American Ale	Wyeast Laboratories
29	Wyeast 2308 Munich Lager	Wyeast Laboratories
30	Wyeast 3068 Weihenstephan Weizen	Wyeast Laboratories

According to the suppliers’ information provided, all ale and lager brewing yeasts (top- and bottom-fermenting strains, respectively) were classified as *Saccharomyces cerevisiae*. One cider yeast strain was provided for comparison (no. 26)

Yeast from one single colony was grown either on liquid yeast extract peptone dextrose (YPD) medium (1 % *w*/*v* Difco Yeast Extract, 2 % *w*/*v* Difco Yeast Bacto-Peptone, 2 % *w*/*v* dextrose) or on solid YPD medium containing 2 % *w*/*v* Difco Bacto agar, at 30 °C.

### Growth rate and cell viability

For the kinetics of growth assay (Lewinska et al. 2011), cells at the logarithmic phase of growth were washed, diluted, suspended in YPD medium, and cultured at 30 °C. Their growth was monitored turbidimetrically at 600 nm in a microplate reader every 2 h during a 10-h period. Cell viability was estimated with a LIVE/DEAD® Yeast Viability Kit (Thermo Fisher Scientific, Poland) using the standard protocol according to the manufacturer’s instructions as described elsewhere (Lewinska et al. 2014a). Briefly, cells at the logarithmic phase of growth were washed and stained with a mixture of FUN® 1 and Calcofluor® White M2R and inspected under an Olympus BX61 fluorescence microscope equipped with a DP72 CCD camera and Olympus CellF software. Typically, a total of 200 cells were used for the analysis.

### Fluorescence-activated cell sorting-based ploidy analysis

The DNA content was measured via flow cytometry as previously described (Deregowska et al. 2015b).

### Pulsed-field gel electrophoresis

Preparation of agarose-embedded yeast DNA and pulsed-field gel electrophoresis (PFGE) separation of yeast DNA were conducted as described elsewhere (Lewinska et al. 2014b). The dendrogram of chromosomal DNA-based similarity was created using Free-Tree software using unweighted pair group method with arithmetic mean (UPGMA) algorithm, Jaccard similarity coefficient, and Java TreeView 1.1.6.r2 (http://jtreeview.sourceforge.net/) (Deregowska et al. 2015b).

### Array-based comparative genomic hybridization

Genomic DNA of selected brewing strains (4, 6, 8, and 9) was labeled with SureTag DNA Labeling Kit and either Cy3- or Cy5-dUTP as previously described (Deregowska et al. 2015b). Briefly, equal amounts of labeled DNA of tested and of the reference laboratory strain (BY4741) were combined and hybridized to Yeast (V2) Gene Expression Microarray, 8x15K using Oligo aCGH Hybridization Kit. All components were supplied by Agilent Technologies Inc. (Santa Clara, CA, USA), and all steps of the experiment were performed according to the manufacturer’s protocols. Following hybridization and washing, the slides were scanned using Axon GenePix 4000B. Feature extraction was conducted using GenePix Pro 6.1 and normalization using Acuity 4.0 (Molecular Devices, Sunnyvale, CA, USA). CGH profiles with superimposed piecewise regression plots to highlight aneuploidies were generated using CGH-Explorer v3.2 (Lingjaerde et al. 2005). The original CGH profiles obtained after the comparison of analyzed strains to BY4741 gave consistently high noise due most probably to genomic DNA sequence differences between BY4741 and brewing strains that influenced the hybridization strength of individual probes. Therefore, to obtain final CGH profiles, the data for each strain were compared to the average of all strains analyzed.

### Gene analysis after array-based comparative genomic hybridization

The analysis of over-representation of functional categories was performed using Cytoscape v. 2.8.2 with BiNGO v. 2.44 plug-in and hypergeometric test using Benjamini and Hochberg false discovery rate (FDR) correction and significance level of 0.05.

### Cluster analysis

The array-CGH data were subjected to complete linkage clustering with Cluster 3.0 software using Euclidean distance similarity metrics (de Hoon et al. 2004) as previously described (Deregowska et al. 2015b).

### Quantitative reverse transcriptase real-time PCR

Yeast cells at the logarithmic phase of growth were harvested and stored at −80 °C until needed. RNA was extracted using a hot acid phenol method. The removal of genomic DNA contamination and complementary DNA (cDNA) synthesis (with 500 ng of total RNA as a template) was performed using RevertAid First Strand cDNA Synthesis Kit (Thermo Fisher Scientific) according to the manufacturer’s protocol. To ensure that RNA had no genomic DNA contamination, control PCR reactions were done without prior reverse transcription. Reactions were done using LightCycler 480 Sybr Green Master Mix and LightCycler Real Time PCR System (Roche) according to the manufacturer’s protocol. Quantitative reverse transcriptase real-time PCR (qRT-PCR) primers (Table 2) were analyzed for specificity and efficiency.

**Table 2:** Primers used for qRT-PCR

Gene	Forward primer	Reverse primer
*ALD2*	TGTTACCGTT CCTTTTGGCG	TGTGAACTGCTTTTGTTTGAAGATAGGT
*FIT3*	GCAGCAGCAA CACCTGGTCT	CAGCAGTGGTGGTTGCAGTG
*AAD10*	CATTGAGGCTTTGAGCATTAAA	ACATTTCGGTGAGAAATGAAGG
*MAL13*	TGAAATTAGAAGCATGGAAT	AGGTTTAGAAATGGGCAGAG
*ALG9*	CTACCATCAGAACCGCATTC	TCCATGATACAGGAGCAAGC

*ALG9* gene was used as a housekeeping gene. The crossing thresholds (CT) were calculated by the second derivative method using the LightCycler Relative Quantification Software and corrected for PCR efficiency, which was between 1.9 and 2.0.

### Comet assay

Yeast spheroplasts were obtained (Lewinska et al. 2014b), and DNA double-strand breaks (DSBs) were assessed by neutral single-cell microgel electrophoresis (comet assay) as described elsewhere (Dworak et al. 2014). The percentage of tail DNA was used as a parameter of DNA damage.

### Oxidative stress parameters

Intracellular reactive oxygen species (ROS) production was measured using 2′,7′-dichlorodihydrofluorescein diacetate (H_2_DCF-DA), and superoxide production was measured using dihydroethidium as described elsewhere (Lewinska et al. 2014a). Oxidative DNA damage as a level of 8-hydroxy-2′-deoxyguanosine (8-OHdG, 8-oxo-dG) was measured using Epigentek EpiQuik 8-OHdG DNA Damage Quantification Direct Kit (Gentaur, Poland) as previously described (Deregowska et al. 2015b).

### Western blotting

For WB analysis, whole-cell extracts were prepared according to Lewinska et al. (2014a). The following primary antibodies were used: anti-Rad1p (1:400), anti-Rap1p (1:400), anti-Fob1p (1:200), anti-Nop1p (1:400), anti-Act1p (1:1000), and anti-Tub1p (1:400) (Santa Cruz, Abcam). The respective proteins were detected after incubation with one of the horseradish peroxidase-conjugated secondary antibodies (1:80,000, 1:100,000 or 1:125,000) (Sigma). The chemiluminescence signals were detected with a Clarity™ Western ECL Blotting Substrate (BIORAD) and a G:BOX imaging system (Syngene, Cambridge, UK).

### Fluorescence in situ hybridization with whole-chromosome painting probes

To detect chromosome I, XI, and XII signals, whole-chromosome painting probes (WCPPs) were used as previously and comprehensively described (Wnuk et al. 2015). Briefly, cells were synchronized, fixed, treated with zymolyase, and subjected to fluorescence in situ hybridization (FISH) procedure using WCPPs. Chromosome-specific signals were counted and presented as a percentage of 100 total cell scores. Moreover, to analyze the nucleolar ribosomal DNA (rDNA) content (chromosome XII-specific signals), ImageJ software http://rsbweb.nih.gov/ij/ was used as described elsewhere (Lewinska et al. 2014b). Briefly, the integrated fluorescence density (green channel) that is the sum of all pixel values within the marked area of each cell analyzed and equivalent to the product of the area and mean gray value was evaluated. The integrated fluorescence density is presented in relative fluorescence units (RFUs).

### Utilization of non-fermentable carbon sources and tolerance to fermentation-associated stress stimuli

The spot assay (the semiquantitative measurement of growth/survival) (Lewinska et al. 2011) was used. To analyze the utilization of non-fermentable carbon sources, several dilutions (1 × 10^7^, 1 × 10^6^, 1 × 10^5^, 1 × 10^4^, and 1 × 10^3^ cells/ml) of a yeast exponential phase culture in a volume of 2 μl were used, inoculated on solid YPG medium (1 % *w*/*v* Difco Yeast Extract, 2 % *w*/*v* Difco Yeast Bacto-Peptone, 2 % *v*/v glycerol) and YPE medium (1 % *w*/*v* Difco Yeast Extract, 2 % *w*/*v* Difco Yeast Bacto-Peptone, 2 % *v*/*v* ethanol) containing 2 % *w*/*v* agar, at 30 °C, and inspected after 48 h. For stress tolerance analysis, yeast cells were grown on standard solid YPD medium in the presence of NaCl, KCl, and sorbitol (0.5, 1, and 1.5 M) and high glucose concentrations (5, 10, and 20 %), ethanol (2.5, 5, and 10 %) or at different temperature conditions (4, 20, 30, 37, and 55 °C). Hydrogen peroxide toxicity was analyzed after 40-min incubation of cells (1 × 10^7^ cells/ml) in the presence of 2, 5, and 10 mM H_2_O_2_ and transfer to solid YPD medium. Typically, the cell growth was inspected after 48 h. Yeast cells grown at 4 °C were inspected after 120 h.

### Statistical analysis

The results represent the mean ± SD from at least three independent experiments. Statistical significance was assessed by one-way ANOVA using GraphPad Prism 5 and with the Tukey’s multiple comparison test.

## Results

### Electrophoretic karyotyping of brewing yeast strains reveals chromosome pattern variability

**Fig. 1:**
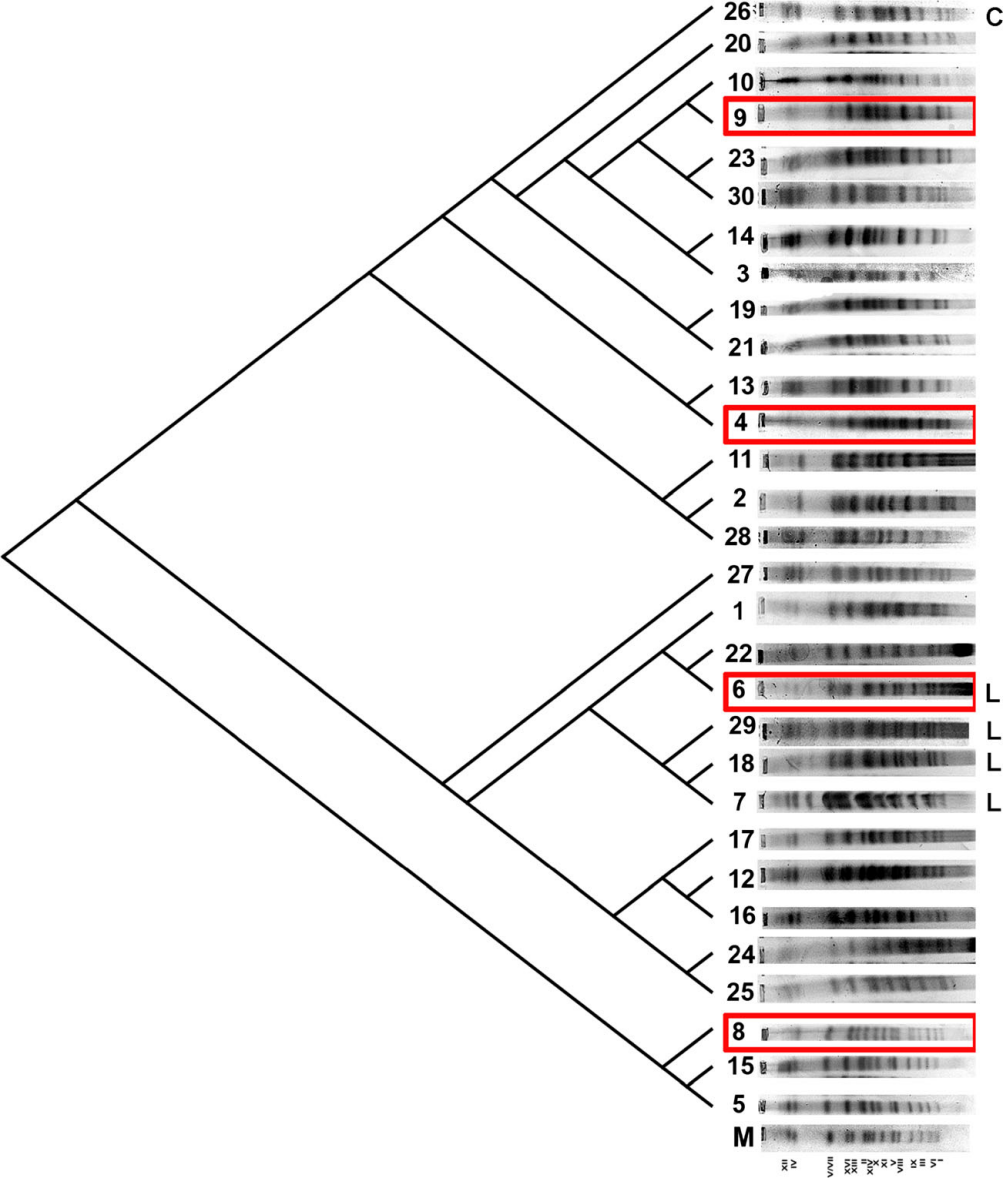
The dendrogram of chromosome band-based similarity created after electrophoretic karyotyping of 30 brewing yeast strains (*lanes* from *1* to *30*). The yeast *S. cerevisiae* chromosome marker YNN295 (BIORAD) is also presented (*lane M*). Lager strains (strains 6, 7, 18, and 29) are denoted as *L*, whereas cider strain (strain 26) is denoted as *C*. Strains 4, 6, 8, and 9 with various chromosome profiles were selected for further analysis (in *red frames*) (Color figure online)

Firstly, we have evaluated the chromosome profiles of 29 brewing yeast strains both top-fermenting ale strains and bottom-fermenting lager strains that were purchased from multiple suppliers and classified as *S. cerevisiae* (Table 1, Fig. 1).

Indeed, after PFGE separation, we were able to observe *S. cerevisiae*-like chromosome profiles (Fig. 1). In general, the chromosome number of analyzed strains is 16 (Fig. 1). However, some additional bands can be also observed; e.g., an additional band between chromosomes IV and VII was shown for strains 6, 7, 18, and 29 that is a characteristic feature of *Saccharomyces bayanus* karyotype (Naumov et al. 1992). Perhaps, some of analyzed strains may be considered as hybrids between *S. cerevisiae* and *S. bayanus.* Indeed, strains 6, 7, 18, and 29 are bottom-fermenting lager strains, and in general, lager yeasts are natural hybrids between *S. cerevisiae* and *S. eubayanus* (Dunn and Sherlock 2008; Walther et al. 2014; Wendland 2014)*.* Some other chromosome variabilities may also reflect increased level of translocations and aneuploidy events and dynamic nature of industrial yeast genomes (Bond et al. 2004; Querol and Bond 2009). Similar chromosome profiles among analyzed strains were revealed using UPGMA clustering; e.g., lager strains were grouped together (Fig. 1). Four strains, namely strains 4, 6, 8, and 9, representing different karyotype profiles were then selected for further analysis (Fig. 1, in red frames). Additionally, one cider yeast strain (strain 26) was included for chromosome comparison (Fig. 1).

### Differences in growth rate, viability, and ploidy state

**Fig. 2:**
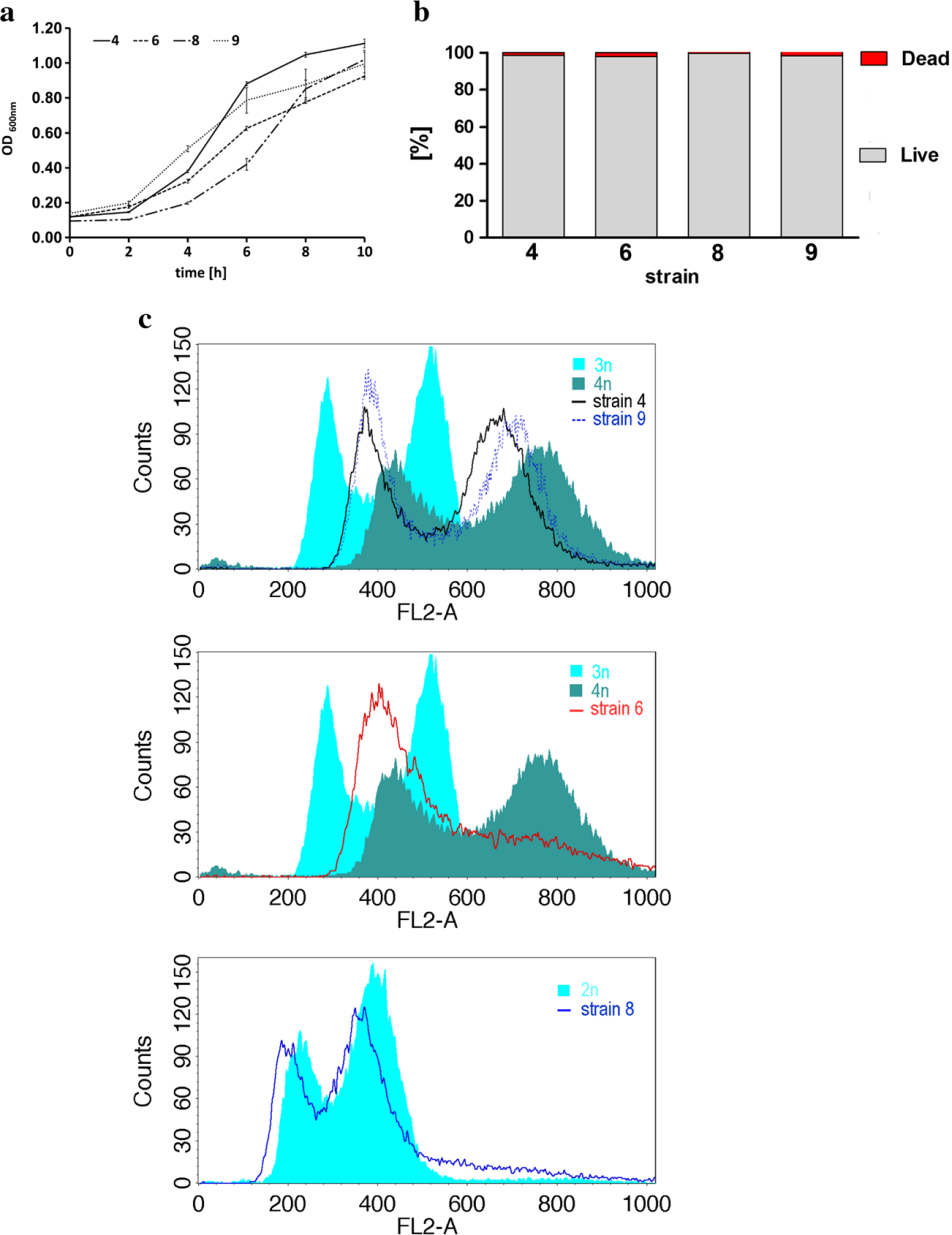
Growth rate, viability, and ploidy state of selected brewing yeast strains. **a** Yeast growth was monitored turbidimetrically at 600 nm in a microplate reader every 2 h during a 10 h. *Bars* indicate SD, *n* = 6. **b** Cell viability was estimated with a LIVE/DEAD® Yeast Viability Kit using the standard protocol according to the manufacturer’s instructions. The percentage of live and dead cells is shown. *n* = 200. **c** Fluorescence-activated cell sorting (FACS)-based analysis of DNA content of strains 4, 6, 8, and 9. Representative histograms are shown. Diploid, triploid, and tetraploid reference strains are also presented

Secondly, the kinetics of growth of selected brewing yeasts both top-fermenting strains (strains 4, 8, and 9) and bottom-fermenting strain (strain 6) was inspected using standard yeast growth medium (YPD medium) (Fig. 2a).

The growth rate of strains 4 and 9 was accelerated compared to the growth rate of strains 6 and 8 in the first 6 h of experiment (Fig. 2a). However, the delay of growth of strains 6 and 8 was overcome in the next 2 h, and after 10 h, the growth yield was comparable between all analyzed strains (Fig. 2a). The viability of cells at the logarithmic phase of growth was similar and ranging from 99.5 to 98 % (Fig. 2b). Fluorescence-activated cell sorting (FACS)-based analysis of DNA content revealed diverse ploidy states of analyzed strains (Fig. 2c). Strains 4 and 9 are tetraploid and strain 8 is diploid. The histogram for strain 6 is more ambiguous and shows some features of the tetraploid reference strain histogram (Fig. 2c).

### Intracellular redox equilibrium is shifted toward more oxidation state in Saflager W-34/70 strain and, to lesser degree, in Windsor British ale strain

**Fig. 3:**
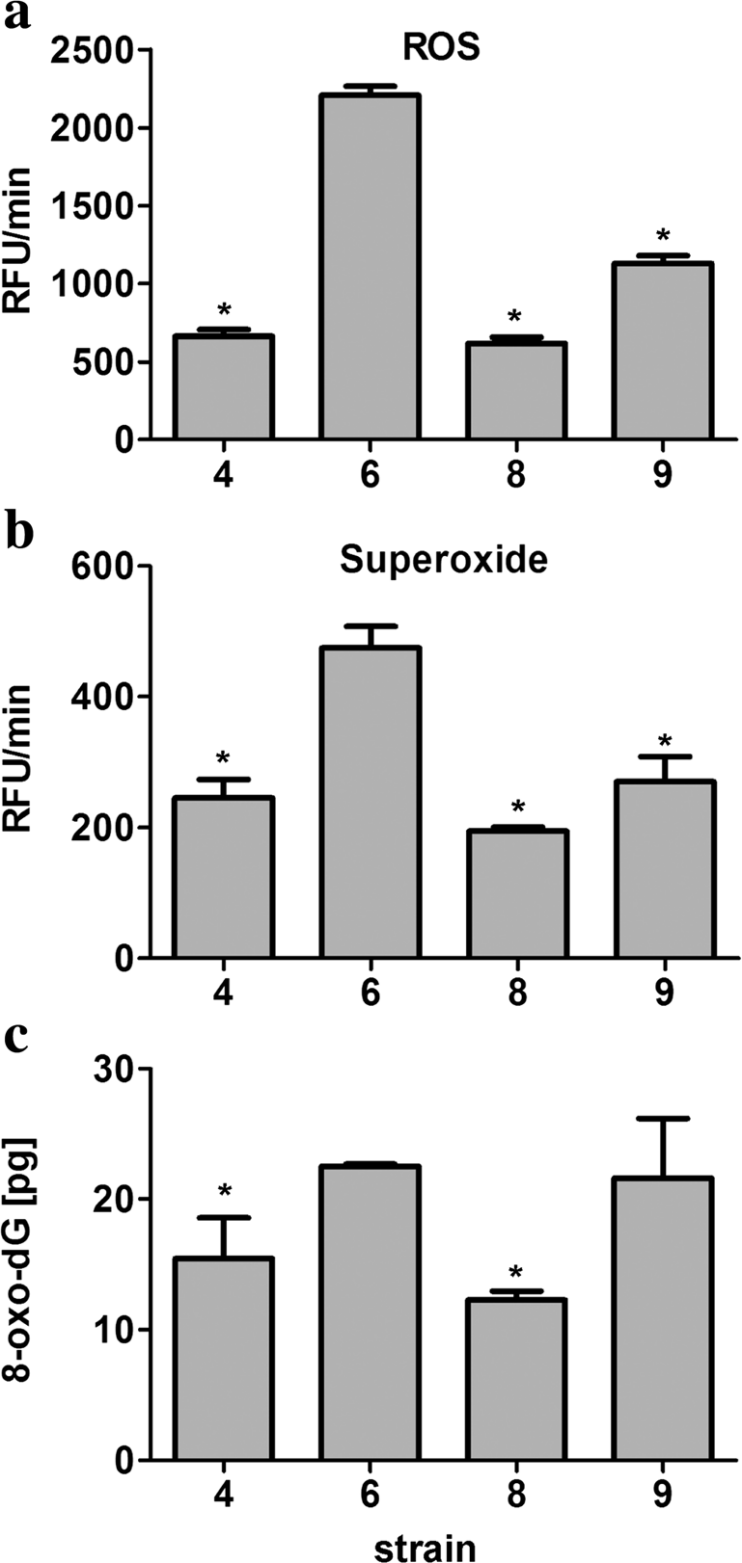
Intracellular redox state of selected brewing yeast strains. Reactive oxygen species (ROS) production was assessed using H_2_DCF-DA fluorogenic probe (**a**), and superoxide production was monitored using dihydroethidium fluorogenic probe (**b**). The results are presented as relative fluorescence units per minute (RFU/min). *Bars* indicate SD, *n* = 4. **c** The level of 8-hydroxy-2′-deoxyguanosine (8-oxo-dG) was analyzed using ELISA-based assay. *Bars* indicate SD, *n* = 3, *p < 0.05 compared to strain 6 (ANOVA and Tukey’s a posteriori test)

We then analyzed intracellular reactive oxygen species (ROS) production and superoxide production in the control growth conditions (Fig. 3).

Redox imbalance was observed in strains 6 and 9 compared to strains 4 and 8 (Fig. 3). Higher level of ROS production of approximately 3.5-fold and 1.8-fold was shown in strains 6 and 9, respectively (Fig. 3a). In contrast, augmented superoxide production was only observed in strain 6 (Fig. 3b). Intracellular superoxide production was higher approximately 2-fold in strain 6 compared to other strains (Fig. 3b). Statistically significant higher level of ROS and superoxide production was observed in strain 6 compared to other strains analyzed (*p* < 0.05) (Fig. 3a, b). Redox disequilibrium resulted in elevated levels of oxidative DNA damage (the level of 8-hydroxy-2′-deoxyguanosine (8-oxo-dG)) in strains 6 and 9 (Fig. 3c). Increased 8-oxo-dG levels of approximately 60 % were observed in strains 6 and 9 compared to strains 4 and 8 (Fig. 3c). Statistically significant higher level of oxidative DNA damage was noticed in strain 6 compared to strains 4 and 8 (*p* < 0.05) (Fig. 3c).

### Subtelomeric regions are sites of the most accented differences in the gene copy number and loci-specific gains and losses

**Fig. 4:**
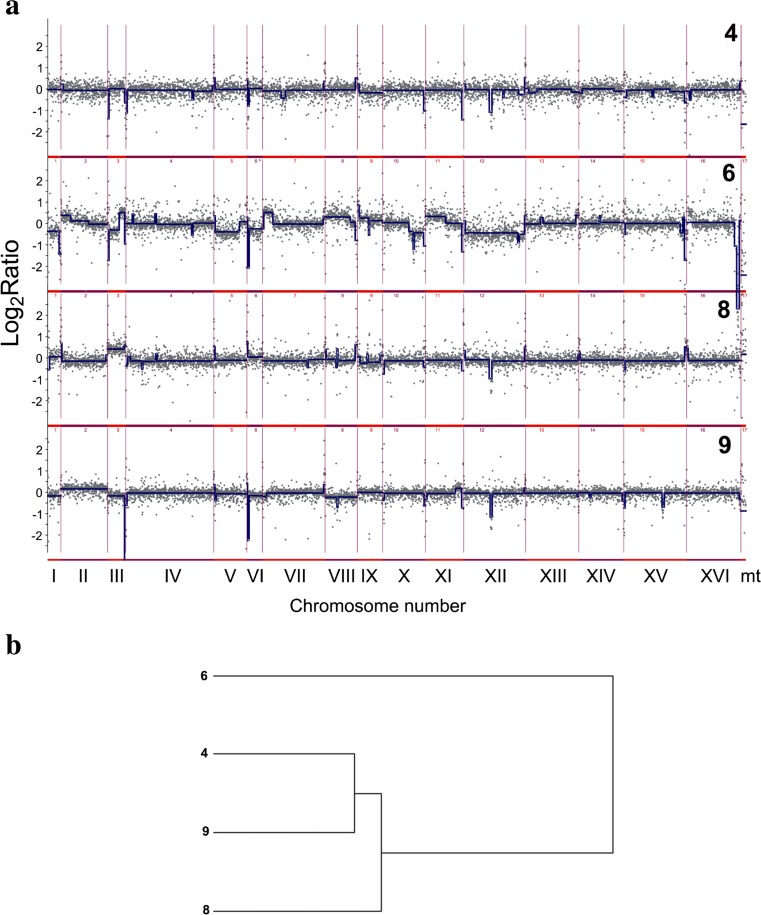
Analysis of the variability in the gene copy number of strains 4, 6, 8, and 9 using array-CGH. **a** Array-CGH profiles are shown. Each *gray dot* represents the value of the log_2_ ratio for an individual gene. *Blue lines* were provided to emphasize the most accented differences (DNA losses and gains). **b** The relatedness of strains analyzed using cluster analysis. Similarity tree is shown (Color figure online)

**Fig. 5:**
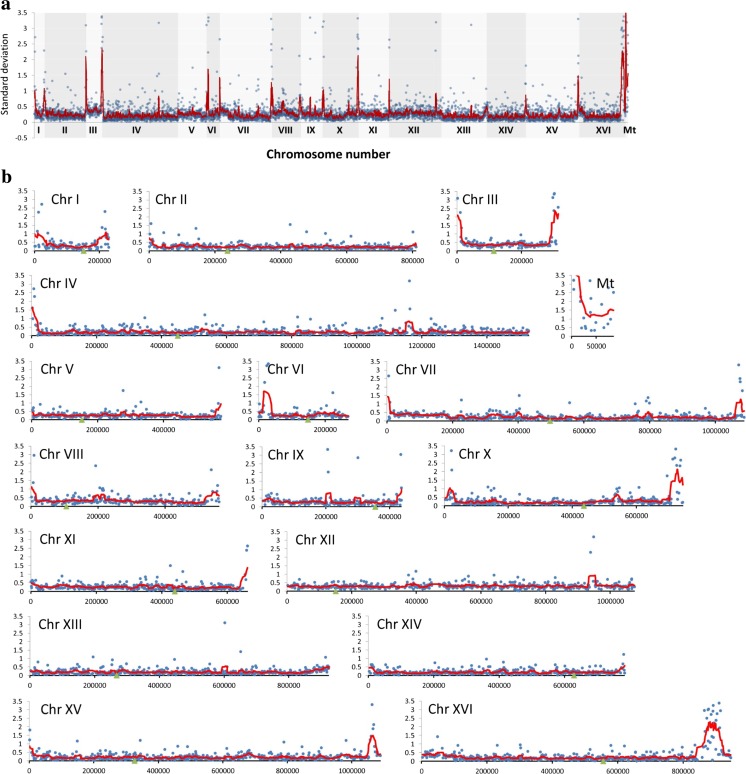
The divergence of relative abundance of genes as determined by array-CGH represented by standard deviation (SD) of log_2_ ratio values for each gene in strains 4, 6, 8, and 9. **a** The summary plot for the whole genome. **b** Individual plots for each chromosome. *Blue dots* indicate the SD values for individual genes, the *red lines* denote the smoother trend calculated by moving average of SD values to expose the genome regions of higher log_2_ ratio divergence, and *green triangles* indicate centromere position. Individual plots for mitochondrial genome are also presented (Mt) (Color figure online)

The genome of selected strains was further characterized using array-based comparative genomic hybridization (array-CGH) (Figs. 4 and 5).

The genome of strain 4 of a euploid nature was characterized by decreased number of subtelomeric genes (Fig. 4a). The gains of chromosomes I, III, and VI were observed in strain 8, whereas the losses of chromosomes I, III, VI, and IX were shown in strain 9 (Fig. 4a). The most affected genome was strain 6 compared to the other analyzed genomes with the majority of its chromosomes containing gains and/or losses (Fig. 4a). The gains of fragments of chromosomes II, III, VII, VIII, and XI and the losses of fragments of chromosomes III, V, and X were observed (Fig. 4a). Moreover, some other losses were much more accented, e.g., gross deficiencies of chromosomes I, VI, XII, and XVI (Fig. 4a). In the case of chromosome XII, the lack of whole chromosome and, in the case of chromosome XVI, the lack of distal part of right arm were observed (Fig. 4a). Additionally, array-CGH profiles were used to estimate the level of similarity (relatedness) between selected strains analyzed on the basis of observed genomic diversity (Fig. 4b). As expected, the most variable was lager strain (strain 6) with its own category (Fig. 4b). All three ale strains were grouped together (Fig. 4b). Array-CGH data allowed us also to analyze the diversity of the copy number of individual genes among all brewing yeast strains studied. As a measure of this diversity, we used standard deviation (SD) of log_2_ ratio values. Figure 5 plots SD of log_2_ ratio values for all genes. Figure 5a gives an overview of the whole yeast genome, and Fig. 5b shows an expanded view of the same data divided into individual chromosomes. The data points for single genes (blue dots) are overplayed with the red line representing the moving average of the individual data points to visualize greater regions of high diversity. According to these plots, the most evident diversity in the gene copy number was revealed within subtelomeric regions in almost all analyzed chromosomes and also within short intrachromosomal regions of chromosomes IV, IX, XII, and XIII (Fig. 5). Also, highly diverse is the whole mitochondrial chromosome (Fig. 5b, Mt).

### Gene ontology overrepresentation profiles vary between strains

**Fig. 6:**
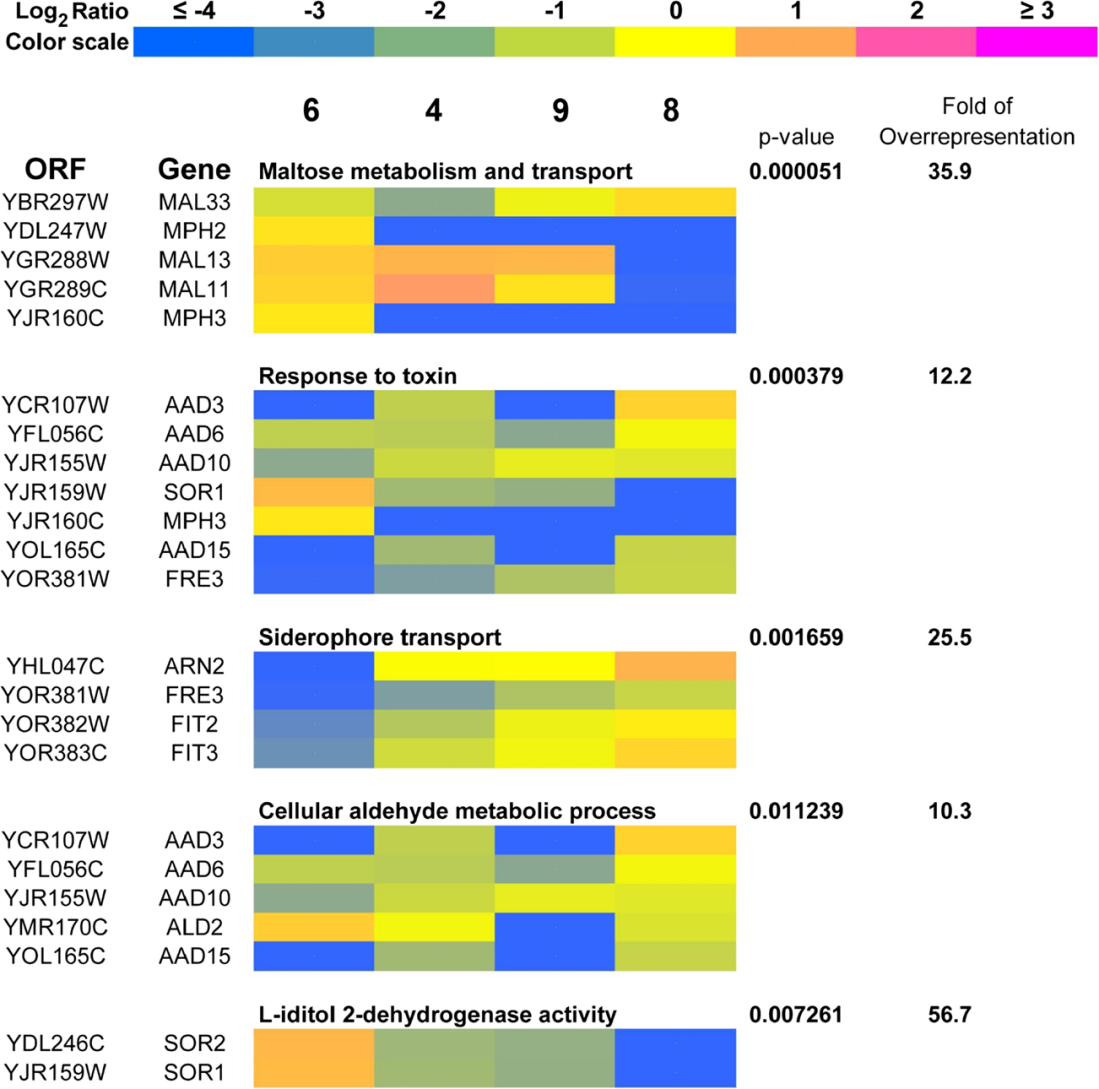
A heat map generated from array-CGH data. Functional categories overrepresented in the group of genes that were the most divergent among analyzed strains are shown. The strains were ordered according to the result of clustering analysis (Fig. 4b), and the selected genes were grouped according to their functional assignment. Positive and negative log_2_ ratio values represent higher and lower than average abundance of the gene, as determined by array-CGH analysis

As the observed differences in the gene copy number and loci-specific gains and losses may affect the functional properties of brewing strains, the genes that were most divergent according to array-CGH-based analysis (showing log_2_ ratio values higher than 2 or lower than −2 for at least one of analyzed strains) were then subjected to gene ontology overrepresentation analysis (Fig. 6).

Five functional categories overrepresented in the group of selected genes were revealed, namely (1) maltose metabolism and transport, (2) response to toxin, (3) siderophore transport, (4) cellular aldehyde metabolic process, and (5) L-iditol 2-dehydrogenase activity (*p* < 0.05) and are presented as a heat map in Fig. 6. Within two functional categories of genes involved in response to toxin and cellular aldehyde metabolic process, the loss of aryl alcohol dehydrogenase (*AAD*) genes was observed in strains 6 and 9 with unbalanced redox equilibrium (Figs. 3 and 6). The effects were statistically significant for *AAD3*, *AAD6*, *AAD10*, and *AAD15* genes (*p* < 0.05) (Fig. 6), whereas similar but weak tendency for *AAD4* and *AAD14* genes was not significant (Supplementary Material). Moreover, the most accented loss of genes involved in siderophore transport was also shown in strain 6 (*p* < 0.05) (Fig. 6). In contrast, the most evident loss of genes involved in maltose metabolism was observed in strain 8 (*p* < 0.05) (Fig. 6). A heat map generated from array-CGH data reflecting the variability in the gene copy number of the whole genome of brewing strains analyzed is also presented in Supplementary Material.

### Validation of array-based comparative genomic hybridization data

To test if the variations in the gene copy number are reflected by the levels of mRNA for those genes, qRT-PCR assay was employed for *FIT3*, *MAL13*, *AAD10*, and *ALD2* genes representing major functional categories depicted in Fig. 6. The qRT-PCR results are presented in Table 3.

**Table 3:** The relative mRNA levels for genes selected from the set shown in Fig. 6

Strain	Gene			
	*FIT3*	*MAL13*	*AAD10*	*ALD2*
4	0.0180 ± 0.0010	1.0668 ± 0.0693	0.2257 ± 0.0417	0.4143 ± 0.0423
6	0.0004 ± 0.0001	0.9243 ± 0.0067	0.0003 ± 0.0001	0.5779 ± 0.0418
8	0.0558 ± 0.0021	0.0004 ± 0.0001	0.4108 ± 0.0084	0.5524 ± 0.0461
9	0.0264 ± 0.0018	0.9239 ± 0.0435	1.3551 ± 0.0274	0.0004 ± 0.0003

The numbers represent the levels of respective transcripts normalized to the data for a housekeeping gene *ALG9*, relative to the normalized levels of transcript in BY4741 strain. The data represent the mean ± SD from at least three independent experiments

**Fig. 7:**
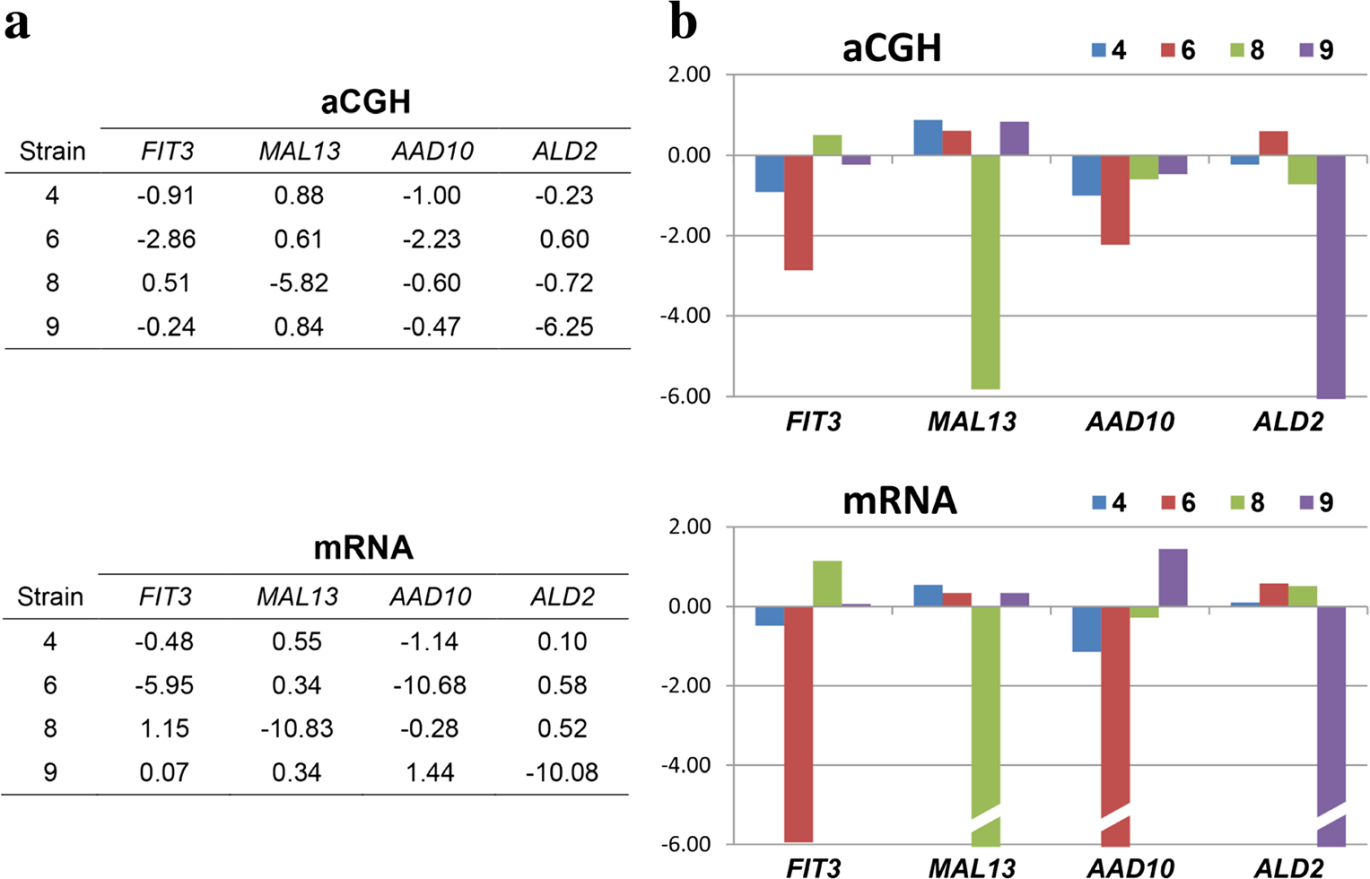
Validation of gene copy number data obtained using array-CGH analysis with mRNA levels determined by qRT-PCR. The comparisons to the array-CGH results were made for genes selected from the set shown in Fig. 6. The qRT-PCR data from Table 3 were normalized with the average of the data for each gene and converted to log_2_ values to bring them to the same format as array-CGH data. The correlation coefficient between both sets of data is 0.89. **a** Log_2_ values obtained with both methods. **b** Graphical representation of these data

Moreover, the comparison of gene copy number and mRNA levels for these genes is shown in Fig. 7.

To make this comparison possible, the qRT-PCR data had to be processed in the same way as array-CGH data. Therefore, the individual data points for each gene were divided by the average of the values for that gene in all strains. The resulting normalized data were converted to log_2_ values. As seen in Fig. 7, the results obtained with both methods correlate very well, with correlation coefficient of 0.89. The negative values obtained for the genes that were absent in some strains are lower using qRT-PCR method than using array-CGH assay, but this is because the former method is more sensitive. Yet, array-CGH result for a gene that is below −2 means that this gene is absent in that strain.

### Genomic stability and nucleolus state are affected in Saflager W-34/70 strain

**Fig. 8:**
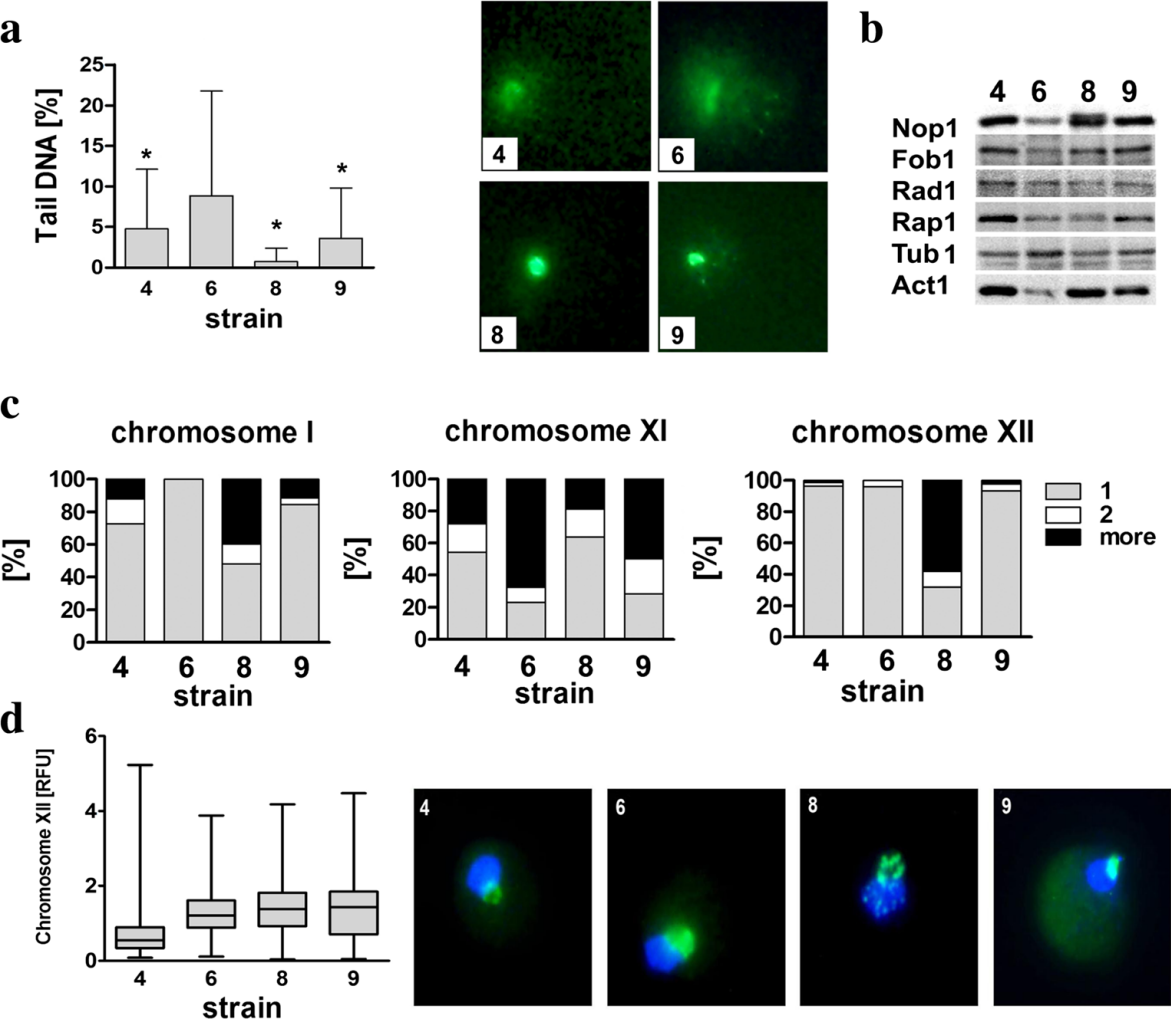
Evaluation of genomic instability and nucleolus state in selected brewing yeast strains in the control growth conditions. **a** The susceptibility to DNA double-strand breaks (DSBs). DSBs were assessed using neutral comet assay. As a DNA damage marker, the % tail DNA was used. The *bars* indicate SD, *n* = 150, *p < 0.05 compared to strain 6 (ANOVA and Tukey’s a posteriori test). The typical micrographs are shown (*right*). DNA was visualized using YOYO-1 staining (*green*). **b** Western blot analysis of Nop1, Fob1, Rad1, and Rap1 contents. Anti-Tub1 antibody served as a loading control. Anti-Act1 antibody was ruled out as a loading control because analyzed strains are characterized by different levels of beta-actin. **c** Analysis of chromosome I, XI, and XII signals using fluorescence in situ hybridization and whole-chromosome painting probes (WCPPs). Chromosome-specific signals were scored in 100 nuclei and presented as a percentage, *n* = 100. Three categories were considered, i.e., cells with one, two, and more than two chromosome specific signals. **d** Analysis of rDNA content. rDNA was visualized using WCPP specific to chromosome XII that contains rDNA locus in yeast. Fluorescence signals of chromosome XII were quantified using ImageJ software. The integrated fluorescence density is presented in relative fluorescence units (RFUs). *Box*-and-*whisker* plots are shown, *n* = 100. The typical micrographs are shown (*right*). The cells were labeled with FITC to detect chromosome XII-specific signals (*green*). DNA was visualized using DAPI staining (*blue*) (Color figure online)

We were then interested if strains with redox disequilibrium and decreased dosage of genes involved in stress responses, e.g., strain 6, may be susceptible to DNA breaks and changes in nucleolus state. Indeed, strain 6 was found to be the most affected by DNA double-strand breaks (DSBs) in the control growth conditions (*p* < 0.05) (Fig. 8a).

We have then compared the levels of protein involved in DNA damage repair, namely Rad1p, but its level was not lower in strain 6 compared to other strains analyzed in the control growth conditions (Fig. 8b). In contrast, the levels of nucleolar proteins Nop1 and Fob1 and transcription regulator Rap1 were lower in strain 6 compared to other strains analyzed in the control growth conditions (Fig. 8b). Interestingly, anti-Act1p antibody cannot be considered as a loading control in strain 6 because strain 6 is characterized by very low level of beta-actin compared to other analyzed strains (Fig. 8b). We selected then three chromosomes of different size, namely small chromosome I, medium-sized chromosome XI, and large chromosome XII to analyze their signal variability in brewing strains using FISH with WCPPs (Fig. 8c). One should remember that array-CGH method is a population-scale approach and is not designed to study the discrete cellular observations, whereas FISH, here single-cell analysis of chromosome instability, can be used to address cellular heterogeneity. Some of our FISH data are in agreement with array-CGH results, especially on genomic diversity observed in strain 6 (Figs. 4 and 8c). Gross deficiencies of chromosomes I and XII (Fig. 4) were revealed using array-CGH that may reflect low frequency of signals of chromosomes I and XII observed using WCPPs (Fig. 8c). Analogically, the gains of chromosome XI (Fig. 4) were correlated with higher frequency of signals of chromosome XI in strain 6 compared to other strains (Fig. 8c). Interestingly, higher frequency of signals of chromosome XII was observed in strain 8 (Fig. 8c, d). Because similar effect was not revealed using array-CGH not detecting rDNA sequences, higher frequency of signals of chromosome XII that contains rDNA locus in yeast may suggest chromosome XII fragmentation and/or nucleolus (rDNA) fragmentation in strain 8. Chromosome XII (rDNA) signals were also quantified (Fig. 8d). However, except of strain 4, rDNA content was comparable among analyzed strains (Fig. 8d). Perhaps, chromosome XII fragmentation does not affect rDNA levels in strain 8 (Fig. 8d).

### Tolerance to fermentation-associated stress stimuli is diminished in Saflager W-34/70 strain

**Fig. 9:**
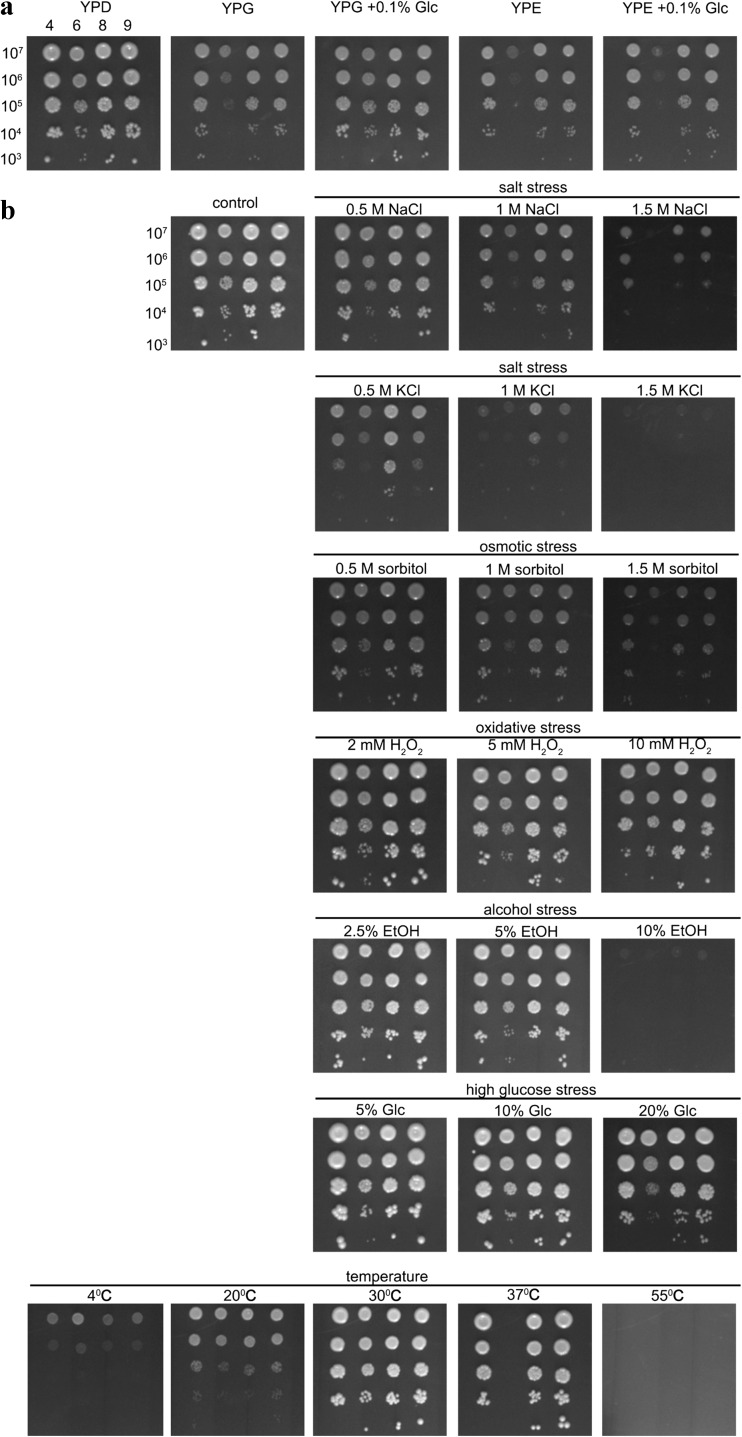
Analysis of the utilization of non-fermentable carbon sources (**a**) and tolerance to fermentation-associated stress stimuli in selected brewing yeast strains (strains 4, 6, 8, and 9) (**b**) using spot assay. **a** Yeast cells at the logarithmic phase of growth were diluted (1 × 10^7^, 1 × 10^6^, 1 × 10^5^, 1 × 10^4^, and 1 × 10^3^ cells/ml), and growth on solid YPG and YPE media was inspected after 48 h. The growth of strain 6 was improved in the presence of 0.1 % glucose in YPG medium. **b** Yeast cells at the logarithmic phase of growth were diluted (1 × 10^7^, 1 × 10^6^, 1 × 10^5^, 1 × 10^4^, and 1 × 10^3^ cells/ml), and growth on solid YPD medium in the presence of different stress stimuli was inspected after 48 h. In the case of hydrogen peroxide, cells were incubated with hydrogen peroxide for 40 min and then transferred to solid YPD medium. The growth of cells incubated at 4 °C was inspected after 120 h. Representative photographs are shown

We then asked the question of whether lower copy number of aryl-alcohol dehydrogenase (*AAD*) genes, imbalanced redox homeostasis, and genetic instability in strain 6 compared to other strains analyzed may also affect fermentation performance in strain 6. First, the utilization of non-fermentable carbon sources, namely glycerol and ethanol, was investigated (Fig. 9a).

The growth capacity of strain 6 was diminished in YPG and YPE media compared to control YPD medium and also to growth of other strains analyzed (Fig. 9a). The growth of strain 6 was improved when YPG medium was supplemented with 0.1 % glucose (Fig. 9a). Second, the tolerance to fermentation-associated stress stimuli was considered, namely salt, osmotic, oxidative, ethanol, high glucose, and cold/heat stresses (Fig. 9b). In general, diminished resistance to stress stimuli of strain 6 was observed compared to other strains analyzed (Fig. 9b). Strain 6 was found to be more sensitive to NaCl, KCl, sorbitol, hydrogen peroxide, ethanol and high-glucose treatments, and heat stress (Fig. 9b). Strain 6 was unable to grow at 37 °C (Fig. 9b). In contrast, cryotolerant lager strain 6 grew better at 4 °C compared to ale strains (strains 4, 8, and 9) (Fig. 9b).

## Discussion

In the present study, we found that the naturally occurring variations in the gene copy number within two functional gene categories: response to toxin and cellular aldehyde metabolic process, mainly the loss of *AAD* genes, may affect intracellular redox equilibrium (elevated ROS production and oxidative DNA damage), the nucleolus state, and the tolerance to fermentation-associated stress stimuli in brewing yeasts of economic importance.

We used 30 commercially available yeast industrial strains, namely 25 ale brewing strains, four lager brewing strains, and one cider strain that, according to the suppliers’ information, belong to *S. cerevisiae* species. Molecular karyotyping revealed the variability of *S. cerevisiae*-like chromosome profiles that reflects genetic and genomic diversity of industrial yeasts (Kodama et al. 2006; Querol and Bond 2009). In the case of lager strains, *S. bayanus*-like chromosomes were also shown that confirm a hybrid nature of lager yeast genome composed of two subgenomes of *S. cerevisiae* and *S. eubayanus* (Dunn and Sherlock 2008; Walther et al. 2014). As ale strains are less studied (U’Ren et al. 2015), we selected three ale brewing yeasts with distinct chromosome patterns for further genomic analysis. One lager strain (Saflager W-34/70), the prototype of lager yeast group II (Frohberg group), was also included for comparison. Although ale yeasts are mostly diploid in nature (Legras et al. 2007), two analyzed ale strains were found to be tetraploid, whereas one strain (strain 8) was denoted as diploid with high variability of chromosome-specific signals (this study). This again confirms a wide range of genomic diversity of brewing yeasts including ale strains (Gonzalez et al. 2008). Whole-genome sequencing revealed that the reference lager yeast group II strain Weihenstephan (WS) 34/70 has approximately 64 chromosomes (4n ploidy with 36 distinct chromosome structures including eight chromosomes with translocations between the two subgenomes) (Nakao et al. 2009; Walther et al. 2014) that is in agreement with our FACS-based analysis of ploidy. Moreover, the analysis of array-CGH profiles showed that the most accented loci-specific gains and losses were observed in Saflager W-34/70 strain compared to other brewing strains.

Statistically significant differences in the gene copy number were revealed in five functional gene categories, namely (1) maltose metabolism and transport, (2) response to toxin, (3) siderophore transport, (4) cellular aldehyde metabolic process, and (5) L-iditol 2-dehydrogenase activity, and analyzed strains varied in gene ontology overrepresentation profiles. We found that decreased dosage of *AAD* genes was correlated with intracellular redox disequilibrium and oxidative DNA damage. In the Saflager W-34/70 (strain 6), the losses of *AAD3*, *AAD6*, *AAD10*, and *AAD15* genes were the most accented and were accompanied by elevated production of reactive oxygen species (ROS) and superoxide, oxidative DNA damage, and diminished tolerance to fermentation-associated stress stimuli, whereas in the Belle Saison Belgian ale strain (strain 8) characterized by the lowest ROS production and the levels of 8-hydroxy-2′-deoxyguanosine (8-oxo-dG), the *AAD* gene set dosage was the highest among analyzed strains. In the *S. cerevisiae* genome, there are seven telomeric open reading frames (ORFs) (YNL331c, YDL243c, YCR107w, YJR155w, YFL056c, YFL057c, and YOL165c) and one non-telomeric ORF (YPL088w) whose protein products show high amino acid sequence similarity to the aryl alcohol dehydrogenase (AAD) of the lignin-degrading fungus *Phanerochaete chrysosporium* (Delneri et al. 1999a). AAD is an enzyme-converting aromatic aldehydes, e.g., veratraldehyde or anisaldehyde, into their corresponding alcohols, and the budding yeast, although not being a lignin degrader, is able to metabolize veratraldehyde into veratryl alcohol (Delneri et al. 1999a). It has been suggested that the telomere-associated members (*AAD3*, *AAD4*, *AAD6*, *AAD10*, *AAD14*, and *AAD15*) form six-member *AAD* gene family and *AAD16* is more distantly related (Delneri et al. 1999b). The expression of the *AAD* genes was shown to be elevated after diamide and diethyl maleic acid ester treatment that promoted oxidative stress by glutathione (GSH) depletion (Delneri et al. 1999b). AAD-mediated oxidative stress response was found to be redox-sensitive transcription factor Yap1 dependent (Delneri et al. 1999b). The genetic analysis using single and multiple *AAD* disruptants revealed that only *AAD4* (YDL243c) and *AAD6* (YFL056/57c) may take part in the oxidative stress response and the contribution of other members to cellular stress responses should be further elucidated (Delneri et al. 1999b). Redox imbalance-mediated susceptibility to oxidative DNA damage of Windsor British ale strain (strain 9) (this study) may also reflect decreased dosage of *ALD2* gene. It has been reported that Ald2p and Ald3p are stress-inducible aldehyde dehydrogenases in *S. cerevisiae* (Navarro-Avino et al. 1999). The expression of *ALD2* and *ALD3* genes is dependent on the general stress transcription factors Msn2,4 (Navarro-Avino et al. 1999). *ALD3* gene expression is induced by osmotic shock, heat shock, glucose exhaustion, oxidative stress, and drugs, whereas *ALD2* gene expression is stimulated by osmotic stress and glucose exhaustion (Navarro-Avino et al. 1999). The double-mutant cells, namely *Δald2Δald3*, are sensitive to ethanol treatment (Navarro-Avino et al. 1999). Moreover, Ald2p and Ald3p are important during acetaldehyde stress in the budding yeast (Aranda and del Olmo 2003). Taken together, the enzymes that participate in NAD^+^/NADH balancing, here AADs and aldehyde dehydrogenases, may be important for the maintenance of intracellular redox homeostasis in brewing yeasts, especially during stresses associated with industrial brewery handling (Gibson et al. 2007).

The other genes characterized by pronounced variations in the gene copy number were genes involved in carbohydrate metabolism and iron transport that may also modulate the fermentation performance. The dosage of maltose utilization genes was affected, namely, *MAL11*, *MAL13*, *MAL33*, *MPH2*, and *MPH3* genes. To metabolize maltose, at least one of five unlinked polymeric (*MAL*) loci located in the telomeric regions of the different chromosomes (*MAL1*-*MAL4*, and *MAL6*) is required (Chow et al. 1989). Decreased dosage of *MAL* genes may not only indicate a defect in maltose fermentation but may also suggest a modulation of drug response pathways as deletions of *MAL11* lead to nystatin sensitivity (Giaever et al. 2002). Analyzed strains varied also in the copy number of *SOR1* and *SOR2* genes that encode a NAD-dependent sorbitol dehydrogenase, a member of the polyol dehydrogenase branch of the medium-chain dehydrogenase/reductase (MDR) superfamily of enzymes (Sarthy et al. 1994). Despite *S. cerevisiae* is a non-xylose-utilizing microorganism, in the presence of sorbitol or xylose, the expression of *SOR1* gene is upregulated (Sarthy et al. 1994; Toivari et al. 2004).

Redox imbalance and lower dosage of *AAD* genes were correlated with DNA breaks and affected nucleolus state (lower levels of Nop1p and Fob1p) in Saflager W-34/70 strain. The nucleolus is suggested to be a guardian of cellular homeostasis and genome integrity acting as a central hub in coordinating the cellular stress response (Grummt 2013). Shifts in the levels and the relocation of nucleolar proteins have been shown during stress conditions both in mammalian and yeast cells that may result in the inhibition of rRNA synthesis saving the energy required to maintain cellular homeostasis during stress (Lewinska et al. 2010; Mayer et al. 2005; Olson 2004; Rubbi and Milner 2003). In contrast, higher levels of Nop1p were correlated with nucleolus (rDNA) fragmentation in strain 8. It has been recently shown that overexpression of other nucleolar protein Nop2 resulted in nucleolus fragmentation (de Beus et al. 1994; Lewinska et al. 2014a). However, rDNA pools were not diminished. Perhaps, living in stressful conditions, industrial strains optimized their growth rate via the control of rDNA level to preserve genome integrity (Deregowska et al. 2015a).

## Conclusions

In summary, we show that strain-specific variability in the gene copy number, especially variations in the dosage of *AAD* genes, may modulate redox homeostasis and susceptibility to DNA damage in brewing yeasts that may be particularly important during fermentation processes when industrial strains are subjected to stress conditions.

## Electronic supplementary material

ESM 1 (XLS 2.20 mb)
